# Redetermination and absolute configuration of pruniflorone M monohydrate

**DOI:** 10.1107/S1600536811025177

**Published:** 2011-07-06

**Authors:** Hoong-Kun Fun, Suchada Chantrapromma, Nawong Boonnak, Chatchanok Karalai, Kan Chantrapromma

**Affiliations:** a X-ray Crystallography Unit, School of Physics, Universiti Sains Malaysia, 11800 USM, Penang, Malaysia; b Crystal Materials Research Unit, Department of Chemistry, Faculty of Science, Prince of Songkla University, Hat-Yai, Songkhla 90112, Thailand; c Research Unit of Natural Products Utilization, Walailak University, Thasala, Nakhon Si Thammarat 80160, Thailand

## Abstract

The title xanthone known as pruniflorone M (systematic name: (2*R*)-5,10-dihy­droxy-2-hy­droxy­methyl-1,1-dimethyl-1*H*-furo[2,3-*c*]xanthen-6-one), crystallized in a monohydrate form, C_18_H_16_O_6_·H_2_O. It was isolated from the green fruits of *Cratoxylum formosum* ssp. *pruniflorum*. The structure of the title compound has been reported previously [Boonnak *et al.* (2010 ▶). *Aust. J. Chem*. **63**, 1550–1556], but we report here the absolute configuration determined using Cu Kα radiation. There are two crystallograpically independent mol­ecules in the asymmetric unit, which differ slightly in the bond angles. The hy­droxy­methyl substituents at position 2 of the furan rings of both pruniflorone M mol­ecules adopt *R* configurations. In both mol­ecules, the three rings of the xanthone skeleton are approximately coplanar, with an r.m.s. deviation of 0.0124 (2) Å for one mol­ecule and 0.0289 (2) Å for the other, and the furan ring adopts an envelope conformation. In the crystal, mol­ecules of pruniflorone M and water are linked into a two-dimensional network by O—H⋯O hydrogen bonds and weak C—H⋯O inter­actions. The crystal structure is further consolidated by π–π inter­actions with centroid–centroid distances in the range 3.5987 (13)–3.7498 (14) Å. Short C⋯C [3.378 (3) Å] and O⋯O [2.918 (3) Å] contacts are also observed.

## Related literature

For details of hydrogen-bond motifs, see: Bernstein *et al.* (1995 ▶) and for ring conformations, see: Cremer & Pople (1975 ▶). For bond-length data, see: Allen *et al.* (1987 ▶). For background to xanthones and their biological activity, see: Boonnak, Karalai *et al.* (2007 ▶); Boonnak *et al.* (2009 ▶, 2010 ▶); Hay *et al.* (2008 ▶); Marques *et al.* (2000 ▶); Molinar-Toribio *et al.* (2006 ▶); Phongpaichit *et al.* (1994 ▶); Yu *et al.* (2007 ▶). For related structures, see: Boonnak *et al.* (2006 ▶); Boonnak, Fun *et al.* (2007 ▶). For the stability of the temperature controller used in the data collection, see Cosier & Glazer (1986 ▶).
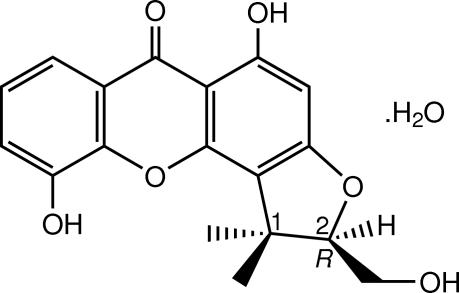


## Experimental

### 

#### Crystal data

C_18_H_16_O_6_·H_2_O
*M* *_r_* = 346.32
Orthorhombic, 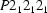

*a* = 9.8887 (3) Å
*b* = 15.6028 (4) Å
*c* = 20.4857 (5) Å
*V* = 3160.77 (15) Å^3^
*Z* = 8
Cu *K*α radiation
μ = 0.95 mm^−1^
*T* = 100 K
0.54 × 0.17 × 0.10 mm

#### Data collection

Bruker APEX DUO CCD area-detector diffractometer
Absorption correction: multi-scan (*SADABS*; Bruker, 2009 ▶) *T*_min_ = 0.627, *T*_max_ = 0.913
13449 measured reflections
4981 independent reflections
4753 reflections with *I* > 2σ(*I*)
*R*_int_ = 0.021

#### Refinement

*R*[*F* ^2^ > 2σ(*F* ^2^)] = 0.043
*wR*(*F* ^2^) = 0.120
*S* = 1.02
4981 reflections
456 parameters
H-atom parameters constrained
Δρ_max_ = 0.56 e Å^−3^
Δρ_min_ = −0.23 e Å^−3^
Absolute structure: Flack (1983 ▶), 2102 Friedel pairs
Flack parameter: 0.06 (19)

### 

Data collection: *APEX2* (Bruker, 2009 ▶); cell refinement: *SAINT* (Bruker, 2009 ▶); data reduction: *SAINT*; program(s) used to solve structure: *SHELXTL* (Sheldrick, 2008 ▶); program(s) used to refine structure: *SHELXTL*; molecular graphics: *SHELXTL*; software used to prepare material for publication: *SHELXTL* and *PLATON* (Spek, 2009 ▶).

## Supplementary Material

Crystal structure: contains datablock(s) global, I. DOI: 10.1107/S1600536811025177/sj5174sup1.cif

Structure factors: contains datablock(s) I. DOI: 10.1107/S1600536811025177/sj5174Isup2.hkl

Supplementary material file. DOI: 10.1107/S1600536811025177/sj5174Isup3.cml

Additional supplementary materials:  crystallographic information; 3D view; checkCIF report

## Figures and Tables

**Table 1:** Hydrogen-bond geometry (Å, °)

*D*—H⋯*A*	*D*—H	H⋯*A*	*D*⋯*A*	*D*—H⋯*A*
O3*A*—H3*OA*⋯O2*A*	0.98	1.62	2.530 (3)	152
O4*A*—H4*OA*⋯O1*WA*	0.92	1.75	2.672 (3)	175
O6*A*—H6*OA*⋯O4*B*^i^	0.82	2.12	2.918 (3)	165
O3*B*—H3*OB*⋯O2*B*	1.06	1.57	2.529 (3)	148
O4*B*—H4*OB*⋯O1*WB*	1.02	1.62	2.639 (3)	175
O6*B*—H6*OB*⋯O4*A*^ii^	0.82	2.25	3.059 (4)	167
O1*WA*—H1*WA*⋯O3*A*^iii^	0.83	2.06	2.889 (3)	173
O1*WA*—H2*WA*⋯O6*B*	0.92	1.84	2.737 (5)	165
O1*WB*—H1*WB*⋯O6*A*	0.89	1.86	2.691 (3)	153
O1*WB*—H2*WB*⋯O3*B*^iv^	0.82	2.06	2.868 (3)	164
C16*B*—H16*C*⋯O2*B*^v^	0.96	2.46	3.389 (3)	163

Symmetry codes: (i) 
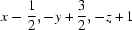
; (ii) 
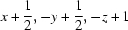
; (iii) 
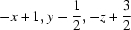
; (iv) 
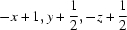
; (v) 
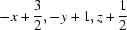
.

**Fig. 1.**
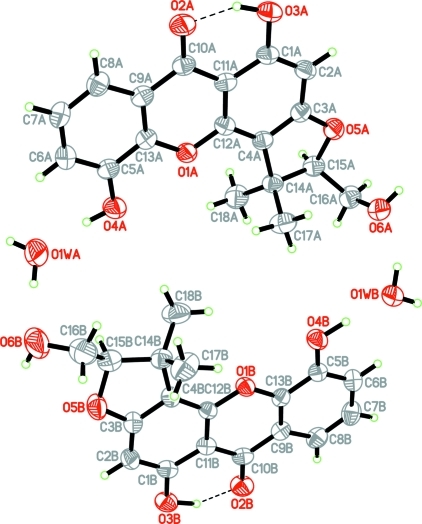
The structure of (I), showing 50% probability displacement ellipsoids and the atom-numbering scheme. Hydrogen bonds are shown as dashed lines.

**Fig. 2.**
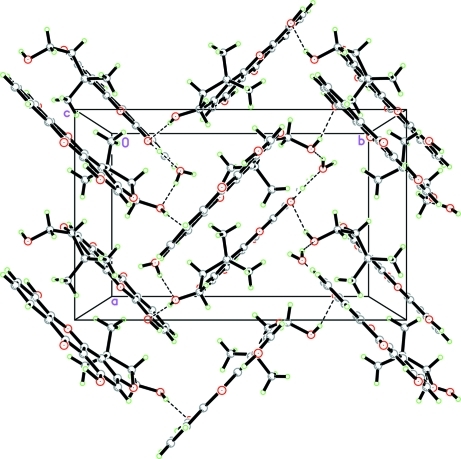
The crystal packing of (I) viewed along the c axis, showing two dimensional network. Hydrogen bonds are shown as dashed lines.

## supplementary crystallographic information

### Comment

Xanthones are secondary metabolites of several plants and exhibit considerable biological activities such as antibacterial, antioxidant, antiprotozoal, cytotoxic and nitric oxide inhibitory activities (Boonnak, Karalai *et al.* 2007; Boonnak *et al.* 2009, 2010; Hay *et al.* 2008; Marques *et al.* 2000; Molinar-Toribio *et al.*, 2006; Phongpaichit *et al.*, 1994; Yu *et al.*, 2007). During the course of our research on the chemical constituents and bioactive compounds from the green fruits of *Cratoxylum formosum* ssp. *pruniflorum*, which were collected from Pha Yao province in the northern part of Thailand, the title xanthone (I) known as pruniflorone M (Boonnak *et al.*, 2010) was isolated. The previous report showed that (I) possess nitric oxide inhibitory activity (Boonnak *et al.*, 2010). The absolute configuration of (I) was determined by making use of the anomalous scattering of Cu Kα*X*-radiation with the Flack parameter being refined to 0.06 (19). We report herein the crystal structure of (I).

There are two crystallograpically independent molecules *A* and *B* in the asymmetric unit of (I), C_18_H_16_O_6_.H_2_O, (Fig. 1) with the same conformation but with slight differences in bond angles. In the structure of (I), the three ring system [C1–C13/O1] is essentially planar with *r.m.s*. deviations of 0.0124 (2) Å for molecule *A* [0.0289 (2) Å for molecule *B*] from the plane through 14 non-hydrogen atoms of the three rings. The O3 and O4 hydroxy O atoms lie close to this plane with deviations +0.038 (2) for O3 and +0.004 (2) Å for O4 for molecule *A* [the corresponding values are +0.043 (2) and -0.024 (2) Å for molecule *B*]. The furan ring (C3–C4/C14–C15/O5) is in an envelope conformation with the puckering atom C15 of 0.148 (3) Å, and puckering parameter Q = 0.239 (2) Å and φ = 132.0 (6)° (Cremer & Pople, 1975) for molecule *A* and the corresponding values are 0.134 (3) Å, 0.213 (3) Å and φ = 137.8 (7)° for molecule *B*. The orientation of the hydroxymethyl moiety at atom C15 can be indicated by the torsion angle of C14–C15–C16–O6 = -73.3 (3)° for molecule *A* [165.0 (3)° for molecule *B*]. Intramolecular O3A—H3OA···O2A and O3B—H3OB..O2B hydrogen bonds (Table 1) generate S(6) ring motifs (Bernstein *et al.*, 1995). The bond distances in (I) are within normal ranges (Allen *et al.*, 1987) and comparable to the related structures (Boonnak *et al.*, 2006; Boonnak, Fun *et al.*, 2007). The hydroxymethyl substituents at position 2 (on atoms C15A and C15B ) of the furan rings of both pruniflorone M  molecules adopt *R* configurations.

In the crystal packing of (I) (Fig. 2), the molecules of pruniflorone M and water are linked into a two dimensional network by O—H···O hydrogen bonds and weak C—H···O interactions (Table 1). π···π interactions were also observed with centroid···centroid distances: *Cg*_1_···*Cg*_5_^v^ = 3.7453 (13) Å; *Cg*_1_···*Cg*_6_^vi^ = 3.6847 (13) Å; *Cg*_2_···*Cg*_4_^vi^ = 3.7189 (12) Å; *Cg*_2_···*Cg*_6_^vi^ = 3.6940 (14) Å; *Cg*_3_···*Cg*_4_^v^ = 3.5987 (13) Å and *Cg*_3_···*Cg*_5_^v^ = 3.7498 (14) Å; *Cg*_1_, *Cg*_2_, *Cg*_3_, *Cg*_4_, *Cg*_5_ and *Cg*_6_ are the centroids of C9A–C13A/O1A, C1A–C4A/C11A–C12A, C5A–C9A/C13A, C9B–C13B/O1B, C1B–C4B/C11B–C12B and C5B–C9B/C13B rings, respectively. C···C^v^[3.378 (3) Å; ] and O···O^i^[2.918 (3) Å short contacts were also observed; [symmetry codes: (i) -1/2+x, 3/2-y, 1-z; (v) 3/2-x, 1-y, 1/2+z and (vi) 1/2-x, 1-y, 1/2-z].

### Experimental

The green fruits of *C. formosum* ssp. *pruniflorum* (5.00 kg) were extracted with CH_2_Cl_2_ (2x20 L, for a week) successively at room temperature and were further evaporated under reduced pressure to afford the crude CH_2_Cl_2_ extracts (31.42 g). The crude extract was further subjected to QCC (Quick Column Chromatography) on silica gel using hexane as a first eluent and then increasing the polarity with acetone to give 14 fractions (F1-F14). Fraction F10 was separated by QCC eluting with a gradient of acetone-hexane to give 17 subfractions (F10A-F10Q). Subfractions F10N was further separated by CC and eluted with a gradient of EtOAc-hexane to give 8 subfractions (F10N1-F10N8). Subfraction F10N2 was further separated by CC and eluted with CHCl_3_ to give the title compound as yellow powder (28.0 mg). Yellow block-shaped single crystals of the title compound suitable for *x*-ray structure determination were recrystallized from CHCl_3_ by the slow evaporation of the solvent at room temperature after several days, Mp. 508-510 K.

### Refinement

All H atoms were placed in calculated positions with (O—H) = 0.82-1.06 Å for OH, (C—H) = 0.93 for aromatic and 0.96 Å for CH_3_ atoms. The *U*_iso_ values were constrained to be 1.5*U*_eq_ of the carrier atom for methyl H atoms and 1.2*U*_eq_ for the remaining H atoms. A rotating group model was used for the methyl groups. The highest residual electron density peak is located at 1.34 Å from O6B and the deepest hole is located at 0.51 Å from O6B. 2102 Friedel pairs were used to determine the absolute configuration. There is no pseudo-symmetry observed in the crystal structure.

### Crystal data

**Table:**

C_18_H_16_O_6_·H_2_O	*D*_x_ = 1.456 Mg m^−^^3^
*M**_r_* = 346.32	Melting point = 508–510 K
Orthorhombic, *P*2_1_2_1_2_1_	Cu *K*α radiation, λ = 1.54178 Å
Hall symbol: P 2ac 2ab	Cell parameters from 4981 reflections
*a* = 9.8887 (3) Å	θ = 5.3–63.5°
*b* = 15.6028 (4) Å	µ = 0.95 mm^−^^1^
*c* = 20.4857 (5) Å	*T* = 100 K
*V* = 3160.77 (15) Å^3^	Block, yellow
*Z* = 8	0.54 × 0.17 × 0.10 mm
*F*(000) = 1456	

### Data collection

**Table:**

Bruker APEX DUO CCD area-detector diffractometer	4981 independent reflections
Radiation source: sealed tube	4753 reflections with *I* > 2σ(*I*)
graphite	*R*_int_ = 0.021
φ and ω scans	θ_max_ = 63.5°, θ_min_ = 5.3°
Absorption correction: multi-scan (*SADABS*; Bruker, 2009)	*h* = −7→11
*T*_min_ = 0.627, *T*_max_ = 0.913	*k* = −18→18
13449 measured reflections	*l* = −23→23

### Refinement

**Table:**

Refinement on *F*^2^	Secondary atom site location: difference Fourier map
Least-squares matrix: full	Hydrogen site location: inferred from neighbouring sites
*R*[*F*^2^ > 2σ(*F*^2^)] = 0.043	H-atom parameters constrained
*wR*(*F*^2^) = 0.120	*w* = 1/[σ^2^(*F*_o_^2^) + (0.0751*P*)^2^ + 0.9688*P*] where *P* = (*F*_o_^2^ + 2*F*_c_^2^)/3
*S* = 1.02	(Δ/σ)_max_ = 0.001
4981 reflections	Δρ_max_ = 0.56 e Å^−^^3^
456 parameters	Δρ_min_ = −0.23 e Å^−^^3^
0 restraints	Absolute structure: Flack (1983), 2102 Friedel pairs
Primary atom site location: structure-invariant direct methods	Flack parameter: 0.06 (19)

### Special details

**Table:**

Experimental. The crystal was placed in the cold stream of an Oxford Cryosystems Cobra open-flow nitrogen cryostat (Cosier & Glazer, 1986) operating at 100.0 (1) K.
Geometry. All esds (except the esd in the dihedral angle between two l.s. planes) are estimated using the full covariance matrix. The cell esds are taken into account individually in the estimation of esds in distances, angles and torsion angles; correlations between esds in cell parameters are only used when they are defined by crystal symmetry. An approximate (isotropic) treatment of cell esds is used for estimating esds involving l.s. planes.
Refinement. Refinement of F^2^ against ALL reflections. The weighted R-factor wR and goodness of fit S are based on F^2^, conventional R-factors R are based on F, with F set to zero for negative F^2^. The threshold expression of F^2^ > 2sigma(F^2^) is used only for calculating R-factors(gt) etc. and is not relevant to the choice of reflections for refinement. R-factors based on F^2^ are statistically about twice as large as those based on F, and R- factors based on ALL data will be even larger.

### Fractional atomic coordinates and isotropic or equivalent isotropic displacement parameters (Å^2^)

**Table:**

	*x*	*y*	*z*	*U*_iso_*/*U*_eq_	
O1A	0.40973 (17)	0.43207 (10)	0.67334 (7)	0.0370 (4)	
O2A	0.3650 (2)	0.44183 (12)	0.87235 (8)	0.0512 (5)	
O3A	0.18470 (19)	0.55552 (12)	0.85896 (8)	0.0483 (4)	
H3OA	0.2444	0.5118	0.8781	0.072*	
O4A	0.58566 (19)	0.32867 (12)	0.61541 (8)	0.0484 (4)	
H4OA	0.6449	0.2869	0.6019	0.062 (9)*	
O5A	0.06909 (18)	0.63151 (11)	0.64104 (8)	0.0444 (4)	
O6A	0.1454 (2)	0.74151 (11)	0.54195 (9)	0.0551 (5)	
H6OA	0.0952	0.7697	0.5655	0.083*	
C1A	0.2000 (2)	0.54898 (15)	0.79348 (11)	0.0357 (5)	
C2A	0.1201 (2)	0.59682 (17)	0.75260 (11)	0.0381 (5)	
H2A	0.0538	0.6334	0.7688	0.046*	
C3A	0.1426 (2)	0.58815 (14)	0.68612 (11)	0.0353 (5)	
C4A	0.2403 (2)	0.53548 (14)	0.65853 (11)	0.0339 (5)	
C5A	0.5798 (2)	0.32706 (15)	0.68149 (11)	0.0378 (5)	
C6A	0.6618 (3)	0.27595 (16)	0.71967 (13)	0.0426 (6)	
H6A	0.7249	0.2404	0.6997	0.051*	
C7A	0.6521 (3)	0.27649 (16)	0.78758 (13)	0.0460 (6)	
H7A	0.7088	0.2418	0.8123	0.055*	
C8A	0.5592 (3)	0.32806 (16)	0.81792 (12)	0.0428 (6)	
H8A	0.5519	0.3275	0.8632	0.051*	
C9A	0.4758 (3)	0.38129 (15)	0.78110 (11)	0.0375 (5)	
C10A	0.3778 (2)	0.43875 (15)	0.81172 (11)	0.0374 (5)	
C11A	0.2983 (2)	0.49098 (15)	0.76823 (11)	0.0341 (5)	
C12A	0.3168 (2)	0.48597 (14)	0.70018 (11)	0.0336 (5)	
C13A	0.4870 (2)	0.38085 (15)	0.71280 (11)	0.0345 (5)	
C14A	0.2429 (2)	0.54925 (16)	0.58514 (11)	0.0376 (5)	
C15A	0.1020 (3)	0.59373 (16)	0.57746 (11)	0.0409 (5)	
H15A	0.0355	0.5485	0.5691	0.049*	
C16A	0.0818 (3)	0.66176 (16)	0.52624 (12)	0.0476 (6)	
H16A	0.1176	0.6411	0.4851	0.057*	
H16B	−0.0144	0.6714	0.5205	0.057*	
C17A	0.3638 (3)	0.6064 (2)	0.56656 (13)	0.0528 (7)	
H17A	0.4466	0.5770	0.5764	0.079*	
H17B	0.3598	0.6589	0.5910	0.079*	
H17C	0.3604	0.6190	0.5207	0.079*	
C18A	0.2474 (3)	0.46718 (18)	0.54526 (13)	0.0535 (7)	
H18A	0.3326	0.4391	0.5520	0.080*	
H18B	0.2370	0.4807	0.4998	0.080*	
H18C	0.1754	0.4299	0.5588	0.080*	
O1B	0.59342 (16)	0.57000 (10)	0.32981 (7)	0.0359 (4)	
O2B	0.64340 (19)	0.55237 (12)	0.13115 (7)	0.0489 (4)	
O3B	0.82577 (19)	0.44097 (12)	0.14837 (8)	0.0470 (4)	
H3OB	0.7592	0.4841	0.1249	0.070*	
O4B	0.42557 (18)	0.67981 (11)	0.38433 (7)	0.0433 (4)	
H4OB	0.3510	0.7205	0.3997	0.065*	
O5B	0.9092 (2)	0.35711 (12)	0.36836 (9)	0.0535 (5)	
O6B	0.9636 (4)	0.2762 (3)	0.49615 (14)	0.1384 (16)	
H6OB	0.9941	0.2549	0.4626	0.208*	
C1B	0.8046 (2)	0.44754 (15)	0.21360 (11)	0.0356 (5)	
C2B	0.8789 (3)	0.39760 (16)	0.25614 (11)	0.0395 (5)	
H2B	0.9461	0.3606	0.2415	0.047*	
C3B	0.8480 (3)	0.40551 (15)	0.32155 (11)	0.0386 (5)	
C4B	0.7515 (2)	0.46058 (14)	0.34767 (11)	0.0353 (5)	
C5B	0.4287 (2)	0.67673 (14)	0.31800 (11)	0.0348 (5)	
C6B	0.3467 (3)	0.72657 (16)	0.27867 (13)	0.0444 (6)	
H6B	0.2848	0.7640	0.2975	0.053*	
C7B	0.3563 (3)	0.72109 (17)	0.21084 (13)	0.0496 (7)	
H7B	0.2996	0.7545	0.1850	0.059*	
C8B	0.4466 (3)	0.66814 (16)	0.18191 (12)	0.0437 (6)	
H8B	0.4534	0.6663	0.1367	0.052*	
C9B	0.5303 (2)	0.61584 (15)	0.22073 (11)	0.0348 (5)	
C10B	0.6281 (2)	0.55735 (15)	0.19175 (10)	0.0359 (5)	
C11B	0.7058 (2)	0.50642 (15)	0.23627 (11)	0.0336 (5)	
C12B	0.6838 (2)	0.51255 (14)	0.30405 (11)	0.0313 (5)	
C13B	0.5192 (2)	0.61998 (14)	0.28842 (11)	0.0328 (5)	
C14B	0.7477 (2)	0.45210 (16)	0.42106 (11)	0.0396 (5)	
C15B	0.8276 (3)	0.36851 (19)	0.42911 (13)	0.0539 (7)	
H15B	0.7623	0.3214	0.4312	0.065*	
C16B	0.9217 (3)	0.35831 (15)	0.48366 (10)	0.0792 (11)	
H16C	0.8832	0.3818	0.5229	0.095*	
H16D	1.0059	0.3875	0.4745	0.095*	
C17B	0.8116 (3)	0.53045 (15)	0.45315 (10)	0.0629 (8)	
H17D	0.9028	0.5369	0.4379	0.094*	
H17E	0.8119	0.5231	0.4997	0.094*	
H17F	0.7604	0.5806	0.4421	0.094*	
C18B	0.6051 (3)	0.4398 (2)	0.44913 (14)	0.0662 (8)	
H18D	0.5559	0.4927	0.4460	0.099*	
H18E	0.6115	0.4230	0.4941	0.099*	
H18F	0.5588	0.3961	0.4249	0.099*	
O1WA	0.7567 (3)	0.21065 (14)	0.56948 (11)	0.0822 (8)	
H1WA	0.7741	0.1639	0.5869	0.123*	
H2WA	0.8243	0.2244	0.5407	0.123*	
O1WB	0.2432 (2)	0.79095 (14)	0.42531 (11)	0.0745 (7)	
H1WB	0.1943	0.7889	0.4620	0.112*	
H2WB	0.2087	0.8328	0.4072	0.112*	

### Atomic displacement parameters (Å^2^)

**Table:**

	*U*^11^	*U*^22^	*U*^33^	*U*^12^	*U*^13^	*U*^23^
O1A	0.0379 (9)	0.0434 (8)	0.0296 (8)	0.0054 (8)	0.0003 (7)	0.0028 (7)
O2A	0.0608 (11)	0.0647 (11)	0.0282 (8)	0.0099 (10)	−0.0001 (8)	0.0017 (8)
O3A	0.0581 (11)	0.0559 (10)	0.0308 (8)	0.0056 (9)	0.0069 (8)	−0.0025 (8)
O4A	0.0507 (10)	0.0568 (10)	0.0378 (9)	0.0077 (9)	0.0027 (8)	0.0010 (8)
O5A	0.0495 (10)	0.0472 (9)	0.0366 (8)	0.0105 (8)	−0.0005 (7)	0.0036 (7)
O6A	0.0697 (13)	0.0465 (9)	0.0491 (10)	0.0035 (9)	0.0140 (9)	0.0032 (8)
C1A	0.0389 (12)	0.0386 (12)	0.0297 (11)	−0.0064 (10)	0.0026 (9)	−0.0022 (10)
C2A	0.0401 (13)	0.0396 (12)	0.0346 (12)	−0.0005 (11)	0.0056 (10)	−0.0039 (9)
C3A	0.0337 (12)	0.0364 (11)	0.0358 (12)	−0.0034 (10)	−0.0022 (10)	0.0030 (10)
C4A	0.0358 (12)	0.0363 (11)	0.0296 (12)	−0.0027 (10)	−0.0005 (9)	−0.0001 (9)
C5A	0.0377 (13)	0.0385 (11)	0.0371 (12)	−0.0047 (11)	0.0006 (10)	0.0021 (10)
C6A	0.0409 (14)	0.0387 (12)	0.0482 (14)	0.0029 (12)	−0.0041 (11)	−0.0003 (11)
C7A	0.0492 (15)	0.0405 (13)	0.0484 (14)	0.0062 (13)	−0.0079 (12)	0.0060 (11)
C8A	0.0475 (15)	0.0425 (12)	0.0385 (13)	−0.0009 (12)	−0.0093 (11)	0.0020 (11)
C9A	0.0388 (13)	0.0396 (13)	0.0341 (12)	−0.0045 (11)	−0.0034 (10)	0.0017 (10)
C10A	0.0368 (13)	0.0439 (12)	0.0314 (11)	−0.0035 (11)	−0.0024 (10)	0.0024 (10)
C11A	0.0351 (12)	0.0376 (11)	0.0297 (11)	−0.0067 (10)	0.0005 (9)	0.0001 (10)
C12A	0.0315 (12)	0.0353 (11)	0.0341 (12)	−0.0043 (10)	0.0012 (10)	−0.0033 (9)
C13A	0.0332 (12)	0.0358 (12)	0.0344 (11)	−0.0043 (10)	−0.0036 (9)	0.0013 (9)
C14A	0.0408 (13)	0.0441 (12)	0.0279 (11)	−0.0001 (11)	−0.0011 (9)	0.0020 (10)
C15A	0.0407 (13)	0.0451 (12)	0.0370 (12)	−0.0020 (11)	−0.0020 (10)	0.0000 (10)
C16A	0.0545 (15)	0.0507 (14)	0.0378 (13)	0.0056 (13)	−0.0074 (12)	0.0036 (11)
C17A	0.0467 (15)	0.0684 (17)	0.0435 (14)	−0.0061 (14)	0.0012 (12)	0.0105 (13)
C18A	0.0688 (19)	0.0559 (15)	0.0358 (13)	0.0049 (14)	−0.0066 (12)	−0.0031 (11)
O1B	0.0369 (9)	0.0433 (8)	0.0274 (7)	0.0066 (7)	0.0002 (6)	−0.0002 (7)
O2B	0.0573 (11)	0.0624 (11)	0.0269 (8)	0.0038 (10)	0.0001 (8)	−0.0015 (8)
O3B	0.0565 (11)	0.0537 (10)	0.0307 (8)	0.0019 (9)	0.0080 (8)	−0.0073 (8)
O4B	0.0482 (10)	0.0480 (9)	0.0339 (8)	0.0089 (8)	0.0033 (8)	−0.0014 (7)
O5B	0.0621 (12)	0.0578 (11)	0.0407 (9)	0.0249 (10)	−0.0030 (9)	0.0005 (8)
O6B	0.152 (3)	0.180 (3)	0.0829 (18)	0.119 (3)	0.0523 (19)	0.066 (2)
C1B	0.0375 (12)	0.0370 (11)	0.0323 (11)	−0.0074 (10)	0.0037 (10)	−0.0076 (9)
C2B	0.0393 (13)	0.0366 (11)	0.0426 (13)	0.0030 (11)	0.0017 (11)	−0.0058 (10)
C3B	0.0419 (13)	0.0361 (11)	0.0379 (12)	0.0043 (10)	−0.0042 (11)	−0.0019 (10)
C4B	0.0383 (12)	0.0343 (11)	0.0333 (12)	−0.0002 (10)	−0.0021 (9)	−0.0045 (9)
C5B	0.0361 (12)	0.0348 (11)	0.0336 (11)	−0.0027 (10)	−0.0001 (10)	−0.0009 (9)
C6B	0.0457 (14)	0.0386 (12)	0.0488 (14)	0.0061 (12)	−0.0013 (12)	−0.0004 (11)
C7B	0.0560 (17)	0.0451 (13)	0.0476 (15)	0.0068 (14)	−0.0128 (13)	0.0047 (12)
C8B	0.0521 (15)	0.0474 (13)	0.0315 (12)	−0.0016 (13)	−0.0073 (11)	0.0034 (11)
C9B	0.0359 (12)	0.0366 (12)	0.0320 (11)	−0.0040 (10)	−0.0048 (9)	0.0002 (9)
C10B	0.0375 (13)	0.0421 (12)	0.0280 (11)	−0.0084 (11)	0.0000 (9)	−0.0020 (10)
C11B	0.0328 (11)	0.0370 (11)	0.0310 (11)	−0.0046 (10)	−0.0012 (9)	−0.0028 (10)
C12B	0.0306 (12)	0.0325 (11)	0.0307 (11)	−0.0011 (9)	0.0015 (9)	−0.0022 (9)
C13B	0.0309 (12)	0.0319 (11)	0.0355 (12)	−0.0020 (10)	−0.0030 (9)	0.0024 (9)
C14B	0.0434 (14)	0.0447 (13)	0.0308 (12)	0.0000 (11)	−0.0027 (10)	0.0013 (10)
C15B	0.0582 (17)	0.0585 (15)	0.0449 (14)	0.0020 (14)	0.0059 (13)	0.0060 (12)
C16B	0.0600 (19)	0.128 (3)	0.0492 (17)	0.033 (2)	−0.0001 (14)	0.0274 (19)
C17B	0.093 (2)	0.0581 (16)	0.0370 (14)	−0.0074 (17)	−0.0121 (15)	−0.0035 (12)
C18B	0.0537 (17)	0.098 (2)	0.0468 (15)	0.0024 (17)	0.0042 (13)	0.0220 (16)
O1WA	0.117 (2)	0.0625 (12)	0.0666 (14)	0.0365 (14)	0.0257 (13)	0.0038 (11)
O1WB	0.0965 (18)	0.0644 (12)	0.0627 (14)	0.0328 (13)	0.0390 (12)	0.0206 (11)

### Geometric parameters (Å, °)

**Table:**

O1A—C12A	1.361 (3)	O2B—C10B	1.253 (3)
O1A—C13A	1.370 (3)	O3B—C1B	1.356 (3)
O2A—C10A	1.249 (3)	O3B—H3OB	1.0564
O3A—C1A	1.354 (3)	O4B—C5B	1.360 (3)
O3A—H3OA	0.9841	O4B—H4OB	1.0223
O4A—C5A	1.355 (3)	O5B—C3B	1.363 (3)
O4A—H4OA	0.9200	O5B—C15B	1.494 (3)
O5A—C3A	1.356 (3)	O6B—C16B	1.371 (4)
O5A—C15A	1.466 (3)	O6B—H6OB	0.8200
O6A—C16A	1.431 (3)	C1B—C2B	1.381 (3)
O6A—H6OA	0.8200	C1B—C11B	1.419 (3)
C1A—C2A	1.372 (3)	C2B—C3B	1.380 (3)
C1A—C11A	1.425 (4)	C2B—H2B	0.9300
C2A—C3A	1.387 (3)	C3B—C4B	1.391 (3)
C2A—H2A	0.9300	C4B—C12B	1.380 (3)
C3A—C4A	1.389 (3)	C4B—C14B	1.510 (3)
C4A—C12A	1.377 (3)	C5B—C6B	1.382 (3)
C4A—C14A	1.519 (3)	C5B—C13B	1.397 (3)
C5A—C6A	1.380 (3)	C6B—C7B	1.395 (4)
C5A—C13A	1.399 (3)	C6B—H6B	0.9300
C6A—C7A	1.394 (4)	C7B—C8B	1.353 (4)
C6A—H6A	0.9300	C7B—H7B	0.9300
C7A—C8A	1.370 (4)	C8B—C9B	1.408 (3)
C7A—H7A	0.9300	C8B—H8B	0.9300
C8A—C9A	1.392 (3)	C9B—C13B	1.393 (3)
C8A—H8A	0.9300	C9B—C10B	1.457 (3)
C9A—C13A	1.404 (3)	C10B—C11B	1.433 (3)
C9A—C10A	1.462 (3)	C11B—C12B	1.409 (3)
C10A—C11A	1.441 (3)	C14B—C17B	1.525 (3)
C11A—C12A	1.408 (3)	C14B—C15B	1.534 (4)
C14A—C18A	1.520 (3)	C14B—C18B	1.535 (4)
C14A—C17A	1.539 (4)	C15B—C16B	1.463 (4)
C14A—C15A	1.565 (3)	C15B—H15B	0.9800
C15A—C16A	1.506 (3)	C16B—H16C	0.9633
C15A—H15A	0.9800	C16B—H16D	0.9669
C16A—H16A	0.9700	C17B—H17D	0.9600
C16A—H16B	0.9700	C17B—H17E	0.9600
C17A—H17A	0.9600	C17B—H17F	0.9600
C17A—H17B	0.9600	C18B—H18D	0.9600
C17A—H17C	0.9600	C18B—H18E	0.9600
C18A—H18A	0.9600	C18B—H18F	0.9600
C18A—H18B	0.9600	O1WA—H1WA	0.8300
C18A—H18C	0.9600	O1WA—H2WA	0.9169
O1B—C13B	1.366 (3)	O1WB—H1WB	0.8939
O1B—C12B	1.371 (3)	O1WB—H2WB	0.8249
			
C12A—O1A—C13A	119.90 (17)	C1B—O3B—H3OB	107.7
C1A—O3A—H3OA	106.0	C5B—O4B—H4OB	110.2
C5A—O4A—H4OA	108.4	C3B—O5B—C15B	106.26 (19)
C3A—O5A—C15A	106.59 (18)	C16B—O6B—H6OB	109.5
C16A—O6A—H6OA	109.5	O3B—C1B—C2B	119.8 (2)
O3A—C1A—C2A	119.9 (2)	O3B—C1B—C11B	118.5 (2)
O3A—C1A—C11A	118.9 (2)	C2B—C1B—C11B	121.7 (2)
C2A—C1A—C11A	121.1 (2)	C3B—C2B—C1B	116.4 (2)
C1A—C2A—C3A	117.0 (2)	C3B—C2B—H2B	121.8
C1A—C2A—H2A	121.5	C1B—C2B—H2B	121.8
C3A—C2A—H2A	121.5	O5B—C3B—C2B	122.4 (2)
O5A—C3A—C2A	122.3 (2)	O5B—C3B—C4B	112.1 (2)
O5A—C3A—C4A	113.02 (19)	C2B—C3B—C4B	125.5 (2)
C2A—C3A—C4A	124.7 (2)	C12B—C4B—C3B	116.5 (2)
C12A—C4A—C3A	117.5 (2)	C12B—C4B—C14B	133.2 (2)
C12A—C4A—C14A	133.1 (2)	C3B—C4B—C14B	110.2 (2)
C3A—C4A—C14A	109.32 (19)	O4B—C5B—C6B	123.3 (2)
O4A—C5A—C6A	123.5 (2)	O4B—C5B—C13B	118.1 (2)
O4A—C5A—C13A	118.3 (2)	C6B—C5B—C13B	118.6 (2)
C6A—C5A—C13A	118.2 (2)	C5B—C6B—C7B	120.4 (2)
C5A—C6A—C7A	121.4 (2)	C5B—C6B—H6B	119.8
C5A—C6A—H6A	119.3	C7B—C6B—H6B	119.8
C7A—C6A—H6A	119.3	C8B—C7B—C6B	121.2 (2)
C8A—C7A—C6A	120.2 (2)	C8B—C7B—H7B	119.4
C8A—C7A—H7A	119.9	C6B—C7B—H7B	119.4
C6A—C7A—H7A	119.9	C7B—C8B—C9B	119.6 (2)
C7A—C8A—C9A	120.1 (2)	C7B—C8B—H8B	120.2
C7A—C8A—H8A	119.9	C9B—C8B—H8B	120.2
C9A—C8A—H8A	119.9	C13B—C9B—C8B	119.3 (2)
C8A—C9A—C13A	119.4 (2)	C13B—C9B—C10B	119.1 (2)
C8A—C9A—C10A	121.7 (2)	C8B—C9B—C10B	121.6 (2)
C13A—C9A—C10A	118.9 (2)	O2B—C10B—C11B	122.1 (2)
O2A—C10A—C11A	122.5 (2)	O2B—C10B—C9B	121.5 (2)
O2A—C10A—C9A	121.1 (2)	C11B—C10B—C9B	116.38 (19)
C11A—C10A—C9A	116.32 (19)	C12B—C11B—C1B	118.2 (2)
C12A—C11A—C1A	118.9 (2)	C12B—C11B—C10B	120.4 (2)
C12A—C11A—C10A	120.6 (2)	C1B—C11B—C10B	121.3 (2)
C1A—C11A—C10A	120.4 (2)	O1B—C12B—C4B	116.81 (19)
O1A—C12A—C4A	117.8 (2)	O1B—C12B—C11B	121.63 (19)
O1A—C12A—C11A	121.5 (2)	C4B—C12B—C11B	121.6 (2)
C4A—C12A—C11A	120.7 (2)	O1B—C13B—C9B	123.3 (2)
O1A—C13A—C5A	116.4 (2)	O1B—C13B—C5B	115.90 (19)
O1A—C13A—C9A	122.8 (2)	C9B—C13B—C5B	120.8 (2)
C5A—C13A—C9A	120.8 (2)	C4B—C14B—C17B	110.42 (19)
C4A—C14A—C18A	114.4 (2)	C4B—C14B—C15B	99.73 (19)
C4A—C14A—C17A	109.87 (19)	C17B—C14B—C15B	115.0 (2)
C18A—C14A—C17A	109.4 (2)	C4B—C14B—C18B	114.0 (2)
C4A—C14A—C15A	98.47 (18)	C17B—C14B—C18B	108.6 (2)
C18A—C14A—C15A	110.2 (2)	C15B—C14B—C18B	109.1 (2)
C17A—C14A—C15A	114.2 (2)	C16B—C15B—O5B	106.2 (2)
O5A—C15A—C16A	107.8 (2)	C16B—C15B—C14B	120.1 (2)
O5A—C15A—C14A	106.66 (19)	O5B—C15B—C14B	106.9 (2)
C16A—C15A—C14A	120.1 (2)	C16B—C15B—H15B	107.7
O5A—C15A—H15A	107.2	O5B—C15B—H15B	107.7
C16A—C15A—H15A	107.2	C14B—C15B—H15B	107.7
C14A—C15A—H15A	107.2	O6B—C16B—C15B	115.9 (3)
O6A—C16A—C15A	113.4 (2)	O6B—C16B—H16C	108.7
O6A—C16A—H16A	108.9	C15B—C16B—H16C	110.2
C15A—C16A—H16A	108.9	O6B—C16B—H16D	102.5
O6A—C16A—H16B	108.9	C15B—C16B—H16D	110.3
C15A—C16A—H16B	108.9	H16C—C16B—H16D	108.9
H16A—C16A—H16B	107.7	C14B—C17B—H17D	109.5
C14A—C17A—H17A	109.5	C14B—C17B—H17E	109.5
C14A—C17A—H17B	109.5	H17D—C17B—H17E	109.5
H17A—C17A—H17B	109.5	C14B—C17B—H17F	109.5
C14A—C17A—H17C	109.5	H17D—C17B—H17F	109.5
H17A—C17A—H17C	109.5	H17E—C17B—H17F	109.5
H17B—C17A—H17C	109.5	C14B—C18B—H18D	109.5
C14A—C18A—H18A	109.5	C14B—C18B—H18E	109.5
C14A—C18A—H18B	109.5	H18D—C18B—H18E	109.5
H18A—C18A—H18B	109.5	C14B—C18B—H18F	109.5
C14A—C18A—H18C	109.5	H18D—C18B—H18F	109.5
H18A—C18A—H18C	109.5	H18E—C18B—H18F	109.5
H18B—C18A—H18C	109.5	H1WA—O1WA—H2WA	109.3
C13B—O1B—C12B	118.98 (17)	H1WB—O1WB—H2WB	100.6
			
O3A—C1A—C2A—C3A	178.9 (2)	O3B—C1B—C2B—C3B	−177.6 (2)
C11A—C1A—C2A—C3A	−2.1 (3)	C11B—C1B—C2B—C3B	2.6 (4)
C15A—O5A—C3A—C2A	−168.9 (2)	C15B—O5B—C3B—C2B	−166.8 (3)
C15A—O5A—C3A—C4A	11.1 (3)	C15B—O5B—C3B—C4B	11.9 (3)
C1A—C2A—C3A—O5A	179.4 (2)	C1B—C2B—C3B—O5B	176.9 (2)
C1A—C2A—C3A—C4A	−0.6 (4)	C1B—C2B—C3B—C4B	−1.6 (4)
O5A—C3A—C4A—C12A	−177.4 (2)	O5B—C3B—C4B—C12B	179.9 (2)
C2A—C3A—C4A—C12A	2.6 (4)	C2B—C3B—C4B—C12B	−1.5 (4)
O5A—C3A—C4A—C14A	5.2 (3)	O5B—C3B—C4B—C14B	2.1 (3)
C2A—C3A—C4A—C14A	−174.8 (2)	C2B—C3B—C4B—C14B	−179.2 (2)
O4A—C5A—C6A—C7A	180.0 (2)	O4B—C5B—C6B—C7B	179.1 (2)
C13A—C5A—C6A—C7A	0.9 (4)	C13B—C5B—C6B—C7B	−1.1 (4)
C5A—C6A—C7A—C8A	0.4 (4)	C5B—C6B—C7B—C8B	−0.9 (4)
C6A—C7A—C8A—C9A	−1.1 (4)	C6B—C7B—C8B—C9B	1.7 (4)
C7A—C8A—C9A—C13A	0.6 (4)	C7B—C8B—C9B—C13B	−0.4 (4)
C7A—C8A—C9A—C10A	−178.5 (2)	C7B—C8B—C9B—C10B	179.5 (2)
C8A—C9A—C10A—O2A	−0.3 (4)	C13B—C9B—C10B—O2B	−178.8 (2)
C13A—C9A—C10A—O2A	−179.4 (2)	C8B—C9B—C10B—O2B	1.3 (4)
C8A—C9A—C10A—C11A	179.5 (2)	C13B—C9B—C10B—C11B	1.0 (3)
C13A—C9A—C10A—C11A	0.4 (3)	C8B—C9B—C10B—C11B	−179.0 (2)
O3A—C1A—C11A—C12A	−178.2 (2)	O3B—C1B—C11B—C12B	179.7 (2)
C2A—C1A—C11A—C12A	2.8 (3)	C2B—C1B—C11B—C12B	−0.5 (3)
O3A—C1A—C11A—C10A	1.4 (3)	O3B—C1B—C11B—C10B	0.9 (3)
C2A—C1A—C11A—C10A	−177.6 (2)	C2B—C1B—C11B—C10B	−179.3 (2)
O2A—C10A—C11A—C12A	179.4 (2)	O2B—C10B—C11B—C12B	−178.7 (2)
C9A—C10A—C11A—C12A	−0.4 (3)	C9B—C10B—C11B—C12B	1.5 (3)
O2A—C10A—C11A—C1A	−0.2 (4)	O2B—C10B—C11B—C1B	0.1 (4)
C9A—C10A—C11A—C1A	180.0 (2)	C9B—C10B—C11B—C1B	−179.7 (2)
C13A—O1A—C12A—C4A	−179.2 (2)	C13B—O1B—C12B—C4B	−176.88 (19)
C13A—O1A—C12A—C11A	0.4 (3)	C13B—O1B—C12B—C11B	2.7 (3)
C3A—C4A—C12A—O1A	177.84 (19)	C3B—C4B—C12B—O1B	−176.83 (19)
C14A—C4A—C12A—O1A	−5.5 (4)	C14B—C4B—C12B—O1B	0.3 (4)
C3A—C4A—C12A—C11A	−1.8 (3)	C3B—C4B—C12B—C11B	3.6 (3)
C14A—C4A—C12A—C11A	174.9 (2)	C14B—C4B—C12B—C11B	−179.2 (2)
C1A—C11A—C12A—O1A	179.6 (2)	C1B—C11B—C12B—O1B	177.8 (2)
C10A—C11A—C12A—O1A	0.0 (3)	C10B—C11B—C12B—O1B	−3.4 (3)
C1A—C11A—C12A—C4A	−0.8 (3)	C1B—C11B—C12B—C4B	−2.7 (3)
C10A—C11A—C12A—C4A	179.6 (2)	C10B—C11B—C12B—C4B	176.1 (2)
C12A—O1A—C13A—C5A	179.80 (19)	C12B—O1B—C13B—C9B	0.0 (3)
C12A—O1A—C13A—C9A	−0.4 (3)	C12B—O1B—C13B—C5B	179.70 (18)
O4A—C5A—C13A—O1A	−0.7 (3)	C8B—C9B—C13B—O1B	178.1 (2)
C6A—C5A—C13A—O1A	178.5 (2)	C10B—C9B—C13B—O1B	−1.8 (3)
O4A—C5A—C13A—C9A	179.5 (2)	C8B—C9B—C13B—C5B	−1.6 (3)
C6A—C5A—C13A—C9A	−1.4 (3)	C10B—C9B—C13B—C5B	178.5 (2)
C8A—C9A—C13A—O1A	−179.2 (2)	O4B—C5B—C13B—O1B	2.4 (3)
C10A—C9A—C13A—O1A	0.0 (3)	C6B—C5B—C13B—O1B	−177.4 (2)
C8A—C9A—C13A—C5A	0.6 (3)	O4B—C5B—C13B—C9B	−177.9 (2)
C10A—C9A—C13A—C5A	179.8 (2)	C6B—C5B—C13B—C9B	2.3 (3)
C12A—C4A—C14A—C18A	48.8 (4)	C12B—C4B—C14B—C17B	−70.3 (3)
C3A—C4A—C14A—C18A	−134.4 (2)	C3B—C4B—C14B—C17B	106.9 (2)
C12A—C4A—C14A—C17A	−74.8 (3)	C12B—C4B—C14B—C15B	168.3 (3)
C3A—C4A—C14A—C17A	102.1 (2)	C3B—C4B—C14B—C15B	−14.4 (3)
C12A—C4A—C14A—C15A	165.6 (3)	C12B—C4B—C14B—C18B	52.2 (4)
C3A—C4A—C14A—C15A	−17.5 (2)	C3B—C4B—C14B—C18B	−130.5 (3)
C3A—O5A—C15A—C16A	−152.6 (2)	C3B—O5B—C15B—C16B	−150.5 (2)
C3A—O5A—C15A—C14A	−22.4 (2)	C3B—O5B—C15B—C14B	−21.1 (3)
C4A—C14A—C15A—O5A	23.6 (2)	C4B—C14B—C15B—C16B	141.8 (2)
C18A—C14A—C15A—O5A	143.6 (2)	C17B—C14B—C15B—C16B	23.8 (3)
C17A—C14A—C15A—O5A	−92.7 (2)	C18B—C14B—C15B—C16B	−98.5 (3)
C4A—C14A—C15A—C16A	146.4 (2)	C4B—C14B—C15B—O5B	20.9 (3)
C18A—C14A—C15A—C16A	−93.5 (3)	C17B—C14B—C15B—O5B	−97.2 (3)
C17A—C14A—C15A—C16A	30.1 (3)	C18B—C14B—C15B—O5B	140.6 (2)
O5A—C15A—C16A—O6A	48.9 (3)	O5B—C15B—C16B—O6B	−73.8 (3)
C14A—C15A—C16A—O6A	−73.3 (3)	C14B—C15B—C16B—O6B	165.0 (3)

### Hydrogen-bond geometry (Å, °)

**Table:**

*D*—H···*A*	*D*—H	H···*A*	*D*···*A*	*D*—H···*A*
O3A—H3OA···O2A	0.98	1.62	2.530 (3)	152
O4A—H4OA···O1WA	0.92	1.75	2.672 (3)	175
O6A—H6OA···O4B^i^	0.82	2.12	2.918 (3)	165
O3B—H3OB···O2B	1.06	1.57	2.529 (3)	148
O4B—H4OB···O1WB	1.02	1.62	2.639 (3)	175
O6B—H6OB···O4A^ii^	0.82	2.25	3.059 (4)	167
O1WA—H1WA···O3A^iii^	0.83	2.06	2.889 (3)	173
O1WA—H2WA···O6B	0.92	1.84	2.737 (5)	165
O1WB—H1WB···O6A	0.89	1.86	2.691 (3)	153
O1WB—H2WB···O3B^iv^	0.82	2.06	2.868 (3)	164
C16B—H16C···O2B^v^	0.96	2.46	3.389 (3)	163

Symmetry codes:  (i) *x*−1/2, −*y*+3/2, −*z*+1; (ii) *x*+1/2, −*y*+1/2, −*z*+1; (iii) −*x*+1, *y*−1/2, −*z*+3/2; (iv) −*x*+1, *y*+1/2, −*z*+1/2; (v) −*x*+3/2, −*y*+1, *z*+1/2.
